# Brachial Plexus Injuries in Adults: Evaluation and Diagnostic Approach

**DOI:** 10.1155/2014/726103

**Published:** 2014-02-09

**Authors:** Vasileios I. Sakellariou, Nikolaos K. Badilas, George A. Mazis, Nikolaos A. Stavropoulos, Helias K. Kotoulas, Stamatios Kyriakopoulos, Ioannis Tagkalegkas, Ioannis P. Sofianos

**Affiliations:** ^1^First Department of Orthopedic Surgery, Athens University Medical School, Attikon University General Hospital, Chaidari, 124 62 Athens, Greece; ^2^Department of Orthopedic Surgery, General Hospital of Levadia, 32100, Levadia, Greece

## Abstract

The increased incidence of motor vehicle accidents during the past century has been associated with a significant increase in brachial plexus injuries. New imaging studies are currently available for the evaluation of brachial plexus injuries. Myelography, CT myelography, and magnetic resonance imaging (MRI) are indicated in the evaluation of brachial plexus. Moreover, a series of specialized electrodiagnostic and nerve conduction studies in association with the clinical findings during the neurologic examination can provide information regarding the location of the lesion, the severity of trauma, and expected clinical outcome. Improvements in diagnostic approaches and microsurgical techniques have dramatically changed the prognosis and functional outcome of these types of injuries.

## 1. Introduction

**Figure 1:**
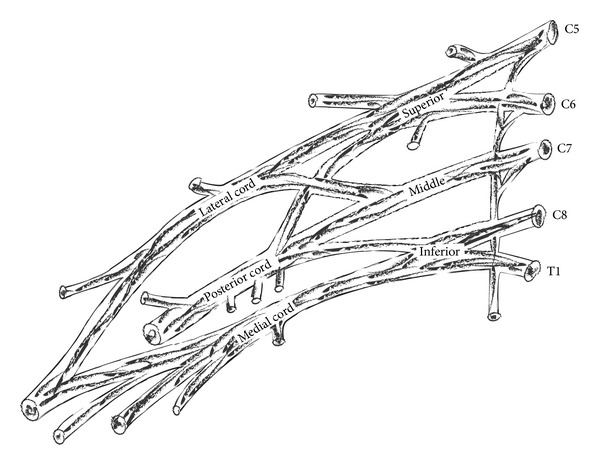
Classic form of brachial plexus.

Brachial plexus is a complex network of nerves, which is responsible for the innervation of the upper extremity. It is formed in the posterior cervical triangle by the union of ventral rami of 5th, 6th, 7th, and 8th cervical nerve roots and 1st thoracic nerve root. This composite nerve network can be divided into roots, trunks, divisions, and cords. The roots, trunks, and divisions lie in the posterior triangle of the neck, whereas the cords lie in the axillary fossa. Cords are further divided in the major nerve branches of the upper extremity [1] (Figure 1).

**Figure 2:**
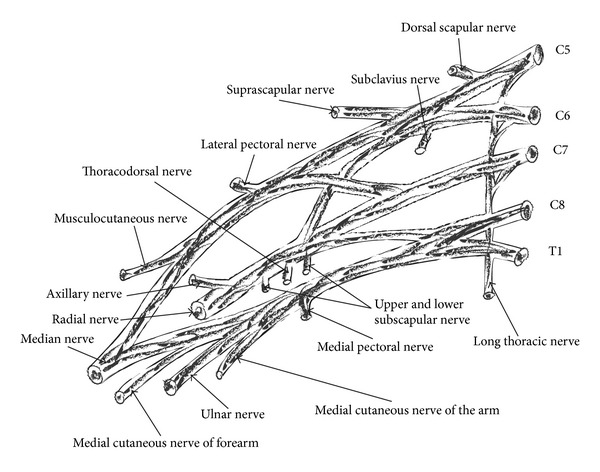
Roots, trunks, divisions, cords, and terminal branches of brachial plexus.

Roots and trunks lie in the supraclavicular space; the divisions are located posterior to the clavicle, while cords and branches lie infraclavicularly [2]. Branches that arise from different portions of brachial plexus are shown in the following figure (Figure 2).

**Figure 3:**
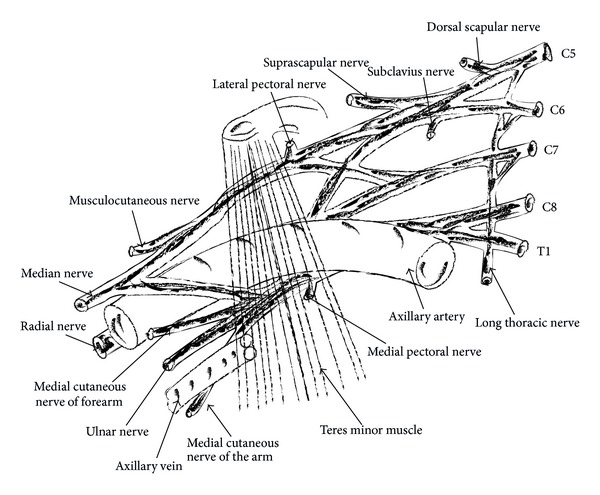
Relationships of brachial plexus and its portions of the axillary artery.

All three cords of the plexus lie above and laterally to the medial portion of axillary artery. Medial cord crosses the artery, passing inferiorly, to reach the medial surface of the middle portion of the artery. Posterior cord is located behind the middle portion of the artery and lateral cord lies laterally to the middle portion of the artery. The names of the cords of brachial plexus imply their relationship to the middle portion of the axillary artery (Figure 3).

**Figure 4:**
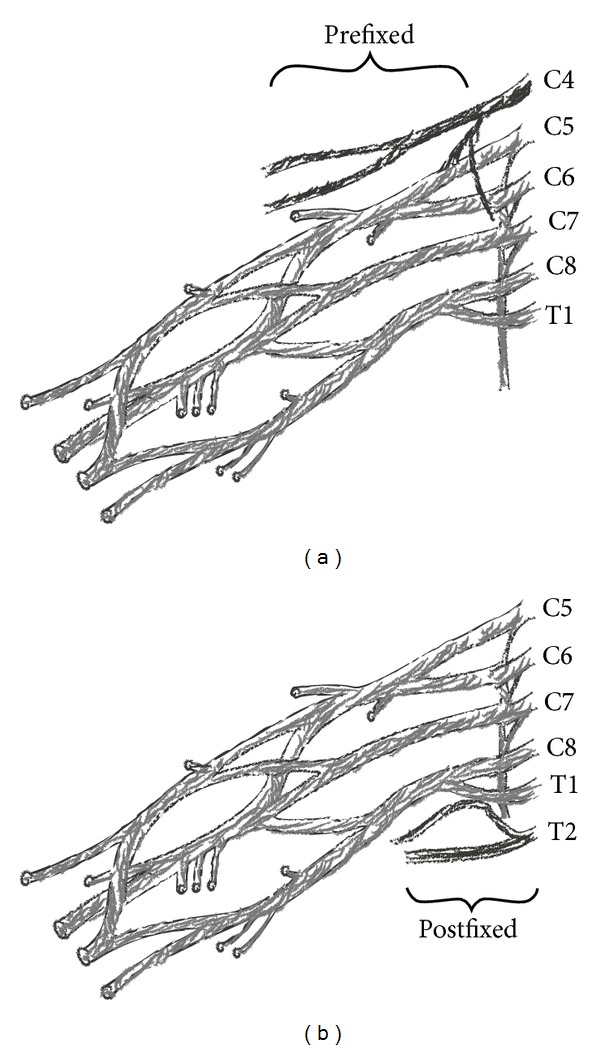
Cephalad (prefixed) and caudal shift (postfixed) of roots that consist of brachial plexus.

Anatomic variants of brachial plexus are observed in more than 50% of the cases [3]. Most common variants are associated with the contribution of C4 (prefixed) or with the contribution of T2 nerve root to the plexus (postfixed) (Figure 4). It is estimated that C4 nerve root contributes to the formation of brachial plexus in 28–62% of cases, according to cadaveric studies [2, 4]. T2 nerve root contributes to the formation of brachial plexus in 16–73% of cases [2, 5, 6]. These contributions may vary in size.

## 2. Pathology

The type of brachial plexus injury depends mainly on the location of injury within the nerve route, that is, within the rootlets, the roots or within the intervertebral foramina through which they pass.

**Figure 5:**
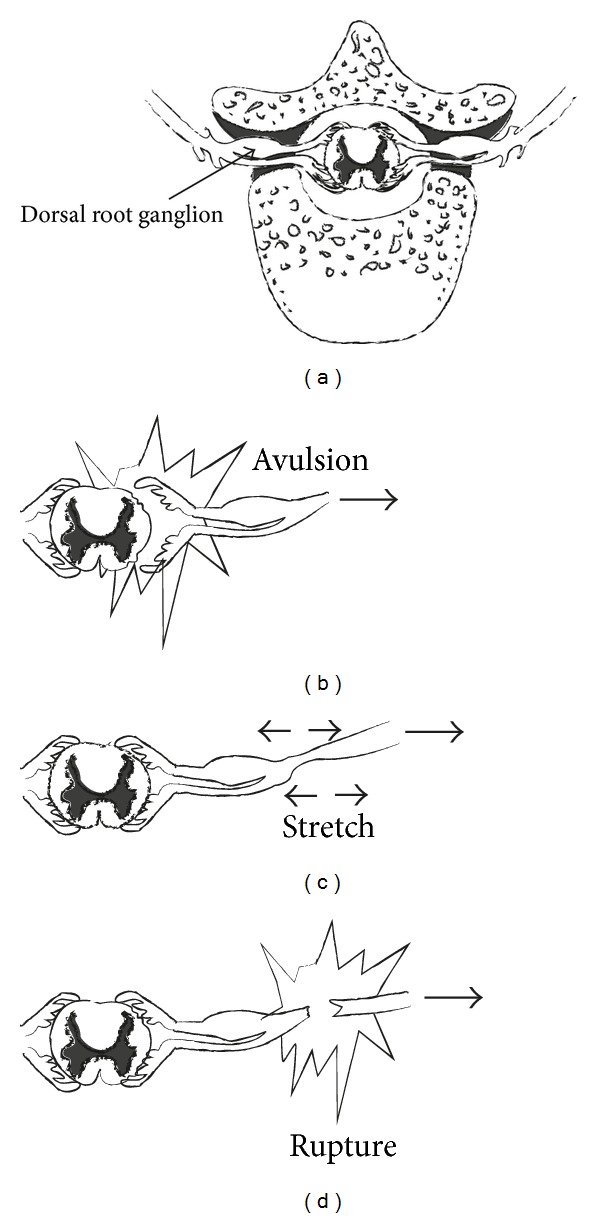
(a) Normal anatomy of rootlets and roots. (b) Avulsive preganglionic injury. (c, d) Postganglionic injury.

In each spinal segment, roots are formed from the union of the dorsal (sensory) and ventral (motor) rootlets that exit the spinal column and pass through the corresponding intervertebral foramen. Cell bodies of sensory neurons are located in the dorsal root ganglia, outside the spinal cord. The rootlets that form cervical roots are located within the spinal cord and lack connective tissue and meningeal coating. As a result to this anatomic peculiarity, rootlets are vulnerable to avulsion injuries from the spinal cord. Spinal nerves have the ability to move freely within the intervertebral foramina, since they do not have any attachments on them. However, there are fibrous attachments that may be found at the C4 to C7 levels, which tie spinal nerves down to transverse processes, after their exit from intervertebral foramina. This does not apply to the spinal nerves of the levels C8 and T1. This anatomical variance explains the comparatively greater incidence of avulsion injuries of the lower two roots of the brachial plexus comparing to the higher anatomic levels [7]. The most important step when examining a brachial plexus injury is to determine whether it affects the roots and is defined as preganglionic (proximal to the dorsal root ganglion) or postganglionic [8] (Figure 5). Determination of the distance of the level of injury from the spinal cord offers very important information. In case of a preganglionic injury, the nerve is avulsed from spinal cord, separating motor neurons from the motor centers of the ventral horns of the spinal cord. Sensory neurons remain intact at the level of dorsal root ganglion, which explains why sensory nerve action potentials are preserved in preganglionic lesions. Preganglionic lesions are not repairable and alternative working motor nerves need to be transferred in order to restore part of the functionality of the upper limp. Contrarily, postganglionic lesions may be restored spontaneously (in case of axonotmesis) or may be repaired surgically. In these lesions, both sensory and motor action potentials are influenced since both sensory and motor rootlets are ruptured.

## 3. Epidemiology

It is difficult to specify the exact number of patients with brachial plexus injuries per year. However, due to the increasing participation in extreme sports and the increased number of motor vehicle accidents survivors, there is global increase in the incidence of brachial plexus injuries [9–14]. There is a significant predilection in male gender and ages between 15 and 25 years old [13, 15–17]. Narakas found that 70% of traumatic brachial plexus lesions are due to traffic accidents and 70% of them involve the use of motorcycles [18].

## 4. Classification of Peripheral Nerves Injuries

Myelinated peripheral nerve fibers are surrounded by Schwann cells. Every nerve fiber with the accompanying Schwann cells is surrounded by a layer of delicate connective tissue, called endoneurium. Groups of nerve fibers compose the fascicles, which are enveloped within a layer of collagen, the perineurium. Most nerves consist of different number of fascicles, which are bundled together by loose collagen fibers, concentrated at the periphery in a relatively thick outer layer, the epineurium.

Historically, peripheral nerve injuries are described by the classification systems of Seddon and Sunderland. The classification proposed by Seddon describes three groups of nerve injuries: neurapraxia, axonotmesis, and neurotmesis [19]. Neurapraxia is defined as the presence of nerve dysfunction without macroscopic lesion of the nerve. Transmission of nerve impulses is interrupted at the site of lesion, which lasts for a short period of time, lasting from a few hours to few months, depending on the area and the severity of the injury. Tinel sign is not elicited during physical examination. Nerve conduction techniques reveal lack of conductivity at the point of the lesion, yet conductivity distally to the injury is normal. This is a pathognomonic finding in neurapraxia [20]. In axonotmesis, the axial continuity of some individual nerve fibers is interrupted, but perineurium and epineurium are preserved. Neurotmesis is defined as a complete disruption of the axon, along with every part of the connective tissue of the peripheral nerve.

Spontaneous recovery of the affected nerve axon cannot be expected. Without surgical intervention, this kind of injury may lead to the creation of a nonfunctional neuroma.

In the classification described by Sunderland, the group of axonotmesis is graded in three degrees, suggesting that different anatomic disruption leads to correspondingly different prognosis [21] (Table 1).

## 5. Mechanism of Injury

**Figure 6:**
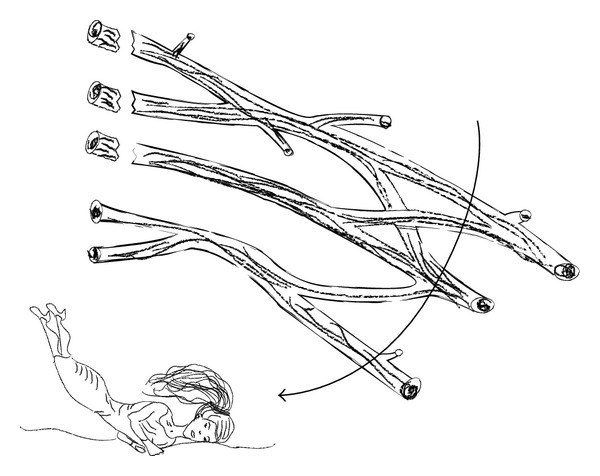
Upper brachial plexus injury occurs when the head and neck are moved away from the ipsilateral shoulder violently.

**Figure 7:**
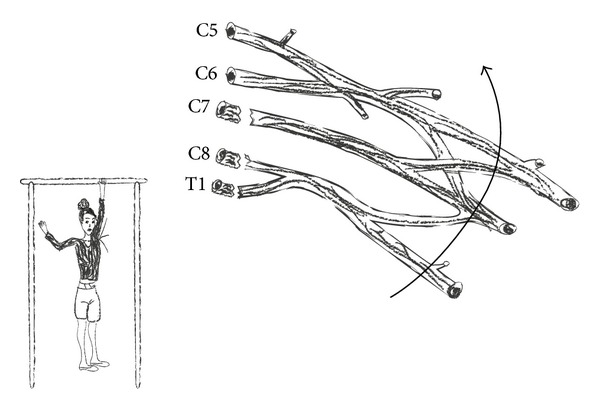
Lower brachial plexus injury occurs when the upper limp is abducted above the level of head with considerable force.

Brachial plexus injuries (BPIs) most commonly affect the supraclavicular zone. Infraclavicular and retroclavicular lesions are less common. Roots and trunks get more easily affected comparing to cords and terminal branches. Two level injuries occur and should be included in differential diagnosis. At the level of supraclavicular region, avulsion injuries are observed after violent lateral head and neck turn away from the ipsilateral shoulder (Figure 6), resulting in the C5, C6, and C7 root or upper trunk disruption. Avulsion injuries can be also observed in the case of forceful traction of the upper limp. When the upper limp is abducted above the level of head with considerable force, avulsion injury of C8-T1 roots or lower trunk is possible (Figure 7). Brachial plexus injuries of the distal part of infraclavicular level are usually caused by high energy trauma in shoulder region. These injuries can be accompanied by rupture of the axillary artery.

Seventy to seventy-five percent of traumatic brachial plexus injuries are located in the supraclavicular region. 75% of them involve total plexus lesions (C5-T1), C5-C6 root injuries account for 20–25% of traumatic BPIs, whereas isolated C8-T1 root lesions account for 2–3.5% of traumatic BPIs. Total brachial plexus injuries usually involve rupture of C5-C6 roots and avulsion of C7-T1 roots [22].

Open brachial plexus injuries may be observed; however, these are much less common comparing to closed BPIs.

Iatrogenic lesions of brachial plexus have been reported during various surgical procedures, including mastectomies, resections of the first rib, and carotid-subclavian bypass operations [23–25].

The outcome of a traumatic brachial plexus injury depends mainly on patient's age, the type of the injured nerve, the level of injury, the time of surgical intervention, and concomitant diseases (Table 2).

## 6. Physical Examination

Brachial plexus injuries are often accompanied by other severe injuries. These injuries may hinder the diagnosis of a nerve injury until patient's recovery. For this reason, high level of suspicion is mandatory in case of an injury of the shoulder girdle or any trauma involving fracture of the first rib or rupture of the axillary artery. Clinically, brachial plexus injuries can be divided according to their location into injuries of the upper plexus (Erb's palsy) and of the lower plexus (Klumpke's palsy). A detailed examination of brachial plexus and its terminal branches can be performed within a few minutes in case of cooperative patients.

In supraclavicular injuries, the shoulder is adducted and internally rotated, whereas the elbow is pronated. Suprascapular nerve injuries, which are located at or posterior to the suprascapular notch, are associated with the presence of tenderness over the notch, muscle weakness during shoulder abduction, and external rotation. Lesions at the level of spinoglenoid notch are related to isolated weakness of infraspinatus muscle [26]. Palsy of long thoracic nerve is clinically evident with a defect during scapular abduction, while dorsal scapular nerve deficit will affect the stabilization of the scapula.

Injuries at the infraclavicular level may have been generated by high energy trauma mechanisms at the shoulder level and may be potentially associated with rupture of the axillary artery. Axillary, suprascapular, and musculocutaneous nerves are most likely the affected nerves at that level of injury [22, 27]. The evaluation of median, ulnar, and radial nerve is performed by the examination of wrist and fingers. The musculocutaneous nerve as well as high lesions of radial nerve is examined by the flexion and extension of the elbow. The axillary nerve, which is a branch of the posterior cord, is examined with active shoulder abduction and deltoid muscle strength. Injury to the posterior cord may affect the function of radial nerve and the muscles that it enervates. Latissimus dorsi is innervated by the thoracodorsal nerve, which is also a branch of the posterior cord and is located closely to the posterior wall of axillary fossa. Pectoralis major receives innervation by the medial and lateral nerves, which are branches of medial and lateral cord, respectively. Lateral anterior thoracic nerve innervates the clavicle, whereas medial anterior thoracic nerve innervates the sternocostal head of the muscle. The muscle can be palpated as the patient adducts his arm under resistance. Proximally, the suprascapular nerve is a terminal nerve branch at the level of trunks.

Motor function of terminal branches is presented in Table 3. Apart from motor dysfunction, sensory deficient is an additional sign to examine.

Clinical findings suggestive of a root avulsion injury are

1. reported constrictive or caustic pain in an otherwise insensitive upper limb;
2. when scapular muscles, serratus anterior, and rhomboid are not functioning, dorsal scapular as well as long thoracic nerve is formed just distally to roots;
3. Horner syndrome-lid ptosis, meiosis (constricted pupil), enopthalmos, and loss of hemifacial sweating (anhidrosis) (T1 root is in proximity to T1 sympathetic ganglion and avulsion injuries occur frequently to both these two structures). Moreover, preganglionic lesions are seldom accompanied by serious vascular injuries, fractures of cervical spine, and dysfunction of spinal cord.

## 7. Diagnostic Approach

### 7.1. Imaging Studies

Radiographic imaging after a neck or shoulder girdle injury may reveal evidence of a concomitant neurological lesion. Radiographs of cervical spine, shoulder girdle, humerus, and chest should be obtained. Cervical spine radiographs should be evaluated for fractures, which could imply that spinal cord is in danger. Moreover, the presence of fracture in the transverse process of a cervical vertebra indicates possible root avulsion at the same level. Clavicle fractures are also indicative of a possible brachial plexus injury. Clavicle radiographs can reveal fractures of the first or second rib, which may suggest injury to the overlying part of brachial plexus.

In addition, a meticulous inspection of a chest radiograph can reveal past rib fractures, which are very important in case that transfer of intercostal nerves is planned, since intercostal nerves are frequently injured by fractures of the corresponding ribs. When phrenic nerve is injured, this is indicated by the presence of elevated and paralyzed hemidiaphragm.

Computed tomography (CT), along with computed tomographic myelography (CTM), contributes greatly to the evaluation of the level of nerve injury [28–30]. In case of a cervical root avulsion a pseudomeningocele could be formed, in the process of dura mater healing. Immediately after the injury, blood clots appear at the avulsion point. These clots become apparent in myelography as overshadowing at the point of the lesion and around. During the first days after the injury, a positive finding may be unreliable, as the dura mater may have been ruptured without concomitant cervical root avulsion. For this reason, CT myelography must be performed 3 to 4 weeks posttraumatically in order to assure that there is enough time for blood clots to be absorbed and for the formation of pseudomeningocele which is an indicative sign of root avulsion injury in CTM.

Myelographic studies in combination with magnetic resonance imaging are essentially a T2 sequence, which points out the contrast of spinal cord and roots to cerebrospinal fluid.

Compared to CT, MRI has certain advantages. It is a noninvasive method which can depict more lesions apart from root injuries and a formed pseudomeningocele. It can depict brachial plexus almost in total. It can reveal posttraumatic neuromas, along with the concomitant inflammatory response and edema of the surrounding tissues.

However, there are a lot of possible mistakes in brachial plexus injuries imagining with conventional MRI. The slices that are taken have relatively large distance between them, in order to reassure acceptable signal to noise ratio (SNRs), with the cost of low resolution and inability to diagnose certain underlying lesions. Moreover, there are artifacts caused by motion such as swallowing, tremor, respiratory, and cardiac movements of the chest, as well as cerebrospinal fluid flow interference which could downgrade the quality of imaging [31].

Even if newer MRI imaging modalities with improved diagnostic results are available, improvements have to be made to reduce artifacts and interferences, in order to establish MRI as a reliable method in detecting brachial plexus root lesions.

### 7.2. Histamine Test

Its purpose is to differentiate preganglionic and postganglionic lesions, but nowadays it is rarely performed. The intradermal injection of histamine causes triple response (red reaction due to capillary dilatation, wheal due to fluid extravasation from increased permeability, and flare due to arteriolar dilatation and to axon reflex in sensory nerve). If there is response with flare in an otherwise unconscious skin area, then the lesion has to be proximal to the dorsal root ganglion, which means that it must be a root avulsion injury. On the contrary, when the lesion is postganglionic the test will be negative as the continuity between the skin and dorsal root ganglion will have been interrupted.

### 7.3. Electrodiagnostic Tests

Electrodiagnostic tests are an integral part of both preoperative and intraoperative evaluation, providing that there is proper conduction and evaluation of their results. Electrodiagnostic evaluation may confirm the diagnosis, pinpoint the lesions, determine the severity of axial discontinuity, and eliminate other clinical entities from differential diagnosis. They are valuable tools, which must be used in conjunction with meticulous physical examination and adequate imaging evaluation, and not as their substitute.

In closed injuries, electromyography (EMG) and nerve conduction velocity studies (NVCs) may be performed 3-4 weeks after the injury, when the conduction of the potentials has stopped along a nerve with postganglionic injury due to Wallerian degeneration. Serial testing in conjunction with repeated physical examination every few months can document and quantify ongoing reinnervation or denervation.

Electromyography (EMG) tests muscles at rest and during activity. Denervation changes (fibrillation potentials) can be seen as early as 10 to 14 days after injury in proximal muscles and as late as 3 to 6 weeks in distal muscles. The presence of voluntary motor unit potentials with limited fibrillation potentials signifies better prognosis than the cases where there is absence of motor unit potentials and many fibrillation potentials.

Nerve conduction velocity studies (NCSs) are performed along with EMG. In posttraumatic brachial plexus injuries, the amplitude of compound muscle action potentials is generally low and is related to the total amount of functional muscle fibers. Sensory nerve action potentials are very important in localizing a lesion as preganglionic or postganglionic. SNAPs will be preserved in lesions proximal to the dorsal root ganglia, due to the fact that the sensory nerve cell bodies are intact within the dorsal root ganglion. For this, in preganglionic injuries there are normal SNAPs in an otherwise insensate dermatome and muscle action potentials (MAPs) are absent. SNAPs will be absent in a postganglionic or combined pre- and postganglionic lesion. Due to the overlapping sensory innervation, especially in the index finger, attention has to be paid when the localization of preganglionic lesion is based on SNAPs exclusively.

It has to be notified that there are obvious restrictions in the diagnostic capacity of electrodiagnostic tests. The diagnostic benefit of EMG/NCSs is related to the experience and the capability of the clinician to interpret the results of these tests. Early signs of muscle recovery may be detected on EMG (occurrence of nascent potentials, decreased number of fibrillation potentials, appearance of or an increased number of motor unit potentials). These signs contribute to expected clinical recovery in weeks or months. However, EMG recovery does not always ensure relevant clinical recovery. In addition, EMG evidence of ongoing reinnervation may not be detected in lesions where target end organs are more distal.

The use of intraoperative electrodiagnostic tests is an integral part of brachial plexus surgery.

Combined electrodiagnostic techniques like nerve action potentials (NAPs), somatosensory evoked potentials (SSEPs), and compound muscle action potentials (CMAPs) may give adequate additional information in order to help the surgeon in decision making [32–34] (Table 4).

## 8. Conclusion

Knowledge of topographic anatomy (origin, course, and relations of the involved roots with the neighboring anatomic elements), anatomy and physiology of central and peripheral nervous system, and connections of roots, trunks, and nerves with target organs, as well as their sensory innervation in combination with repeated, thorough, and recorded clinical examination, are the keystones of diagnostic evaluation of brachial plexus injuries.

Plain radiographs, computed tomography, CT myelography, and magnetic resonance imaging with or without contrast and electrodiagnostic tests are invaluable accessory tools in the evaluation and diagnostic approach of traumatic brachial plexus injuries, each with its own sensitivity and specificity. Each of these techniques plays an important part in intraoperative and postoperative diagnosis and evaluation. In order to optimize the anticipated results, the use of each diagnostic method within the appropriate time frame is of paramount importance.

## Figures and Tables

**Table 1:** Comparison of Seddon and Sunderland classifications of nerve injury.

Sunderland	Seddon	Histopathology
1	Neurapraxia	Functional but not anatomical disorganization
2	Axonotmesis	Intact endoneurium and perineurium
3	Axonotmesis	Intact perineurium
4	Axonotmesis	Intact epineurium
5	Neurotmesis	Disruption of all layers

**Table 2:** Factors that affect the prognosis of a peripheral nerve injury.

Factor	Result
Mechanism of injury	High energy injuries have worse prognosis Avulsion injuries have worse prognosis than acute ruptures Worse prognosis with concomitant vascular injury

Age	Better prognosis in young patients

Type of nerve	Exclusively sensory or motor nerves have better functional recovery than mixed nerves

Level of injury	Supraclavicular lesions have worse prognosis than infraclavicular Upper trunk lesions have the best prognosis

Pain	Patients with persistent pain for more than 6 months after traumatic BPI have less possibilities for recovery

Time of surgical intervention	Fibrosis and degeneration of target organs at the time of surgical intervention are related to poor prognosis

Other factors	Concomitant diseases (infections, etc.) are related to worse prognosis

**Table 3:** Terminal branches of brachial plexus and their action.

Nerve	Muscle	Action
Dorsal scapular (C5)	Rhomboid	Stabilization of scapula

Long thoracic (C5)	Serratus anterior	Abduction of scapula

Suprascapular (C5)	Supraspinatus Infraspinatus	Abduction of shoulder External rotation of shoulder

Medial (C8) and lateral pectoral (C7)	Pectoralis major	Adducts the shoulder
	Pectoralis minor	Stabilizes the scapula

Subscapular (C5)	Subscapularis and teres major	Internal rotation of shoulder

Thoracodorsal (C7)	Latissimus dorsi	Adduction of shoulder

Musculocutaneous (C5)	Biceps brachii and brachialis	Flexion of elbow

Ulnar (C8, T1)	Flexor carpi ulnaris Intrinsic muscles of hand	Flexion of wrist and fingers Abduction of fingers

Median (C6, C7, C8, T1)	Pronators of forearm Flexors of wrist and fingers	Pronation of forearm Flexion of wrist and fingers

Radial (C6, C7, C8)	Supinator Triceps brachii Extensors of wrist and fingers	Supination of forearm Extension of elbow, wrist, and fingers

Axillary (C5)	Deltoid and teres minor	Abduction of shoulder

**Table 4:** Neurophysiological findings in traumatic peripheral nerve injuries, in each category, respectively, according to Seddon classification.

	Neurapraxia	Axonotmesis	Neurotmesis
Conduction velocity	Usually normal	Normal/mild reduction	Absent
CMAP frequency	Normal/reduced	Reduced	Absent
SNAP frequency	Reduced	Reduced	Absent
Abnormal spontaneous potentials in EMG	Absent	Probably present	Present

Source: [8].

## References

[1] Snell RS (2007). *Clinical Anatomy*.

[2] Kerr A (1918). Brachial plexus of nerves in man. The variations in its formation and branches. *American Journal of Anatomy*.

[3] Uysal II, Şeker M, Karabulut AK (2003). Brachial plexus variations in human fetuses. *Neurosurgery*.

[4] Senecail B (1975). *Le plexus brachial de l’Homme [Ph.D. thesis]*.

[5] Adolphi H (1898). Uber das Verhalten der zweiten Brustnerven zum plexus brachialis beim Menschen. *Anatomischer Anzeiger*.

[6] Hirasawa K (1927). Uber den pelxus brachialis mitterlung die wurzeln des plexus brachial. *Impressio Separata ex Actis Scholac Medicinalis*.

[7] Shin AY, Spinner RJ (2005). Clinically relevant surgical anatomy and exposures of the brachial plexus. *Hand Clinics*.

[8] Gregory J, Cowey A, Jones M, Pickard S, Ford D (2009). The anatomy, investigations and management of adult brachial plexus injuries. *Orthopaedics and Trauma*.

[9] Allieu Y, Cenac P (1988). Is surgical intervention justifiable for total paralysis secondary to multiple avulsion injuries of the brachial plexus?. *Hand Clinics*.

[10] Azze RJ, Mattar R, Ferreira MC, Starck R, Canedo AC (1994). Extraplexual neurotization of brachial plexus. *Microsurgery*.

[11] Brandt KE, Mackinnon SE (1993). A technique for maximizing biceps recovery in brachial plexus reconstruction. *Journal of Hand Surgery*.

[12] Brunelli G, Monini L (1985). Direct muscular neurotization. *Journal of Hand Surgery*.

[13] Doi K, Muramatsu K, Hattori Y (2000). Restoration of prehension with the double free muscle technique following complete avulsion of the brachial plexus. Indications and long-term results. *Journal of Bone and Joint Surgery A*.

[14] Doi K, Kuwata N, Muramatsu K, Hottori Y, Kawai S (1999). Double muscle transfer for upper extremity reconstruction following complete avulsion of the brachial plexus. *Hand Clinics*.

[15] Malone JM, Leal JM, Underwood J, Childers SJ (1982). Brachial plexus injury management through upper extremity amputation with immediate postoperative prostheses. *Archives of Physical Medicine and Rehabilitation*.

[16] Allieu Y (1999). Evolution of our indications for neurotization. Our concept of functional restoration of the upper limb after brachial plexus injuries. *Annales de Chirurgie de la Main et du Membre Superieur*.

[17] Dubuisson AS, Kline DG, Amar AP, Gruen JP, Kliot M, Yamada S (2002). Brachial plexus injury: a survey of 100 consecutive cases from a single service. *Neurosurgery*.

[18] Narakas AO (1985). The treatment of brachial plexus injuries. *International Orthopaedics*.

[19] Seddon HJ (1943). Three types of nerve injury. *Brain*.

[20] Mackinnon SE, Goldwyn RM, Cohen MN (2001). Nerve grafts. *The Unfavorable Result in Plastic Surgery*.

[21] Sunderland S (1951). A classification of peripheral nerve injuries producing loss of function. *Brain*.

[22] Moran SL, Steinmann SP, Shin AY (2005). Adult brachial plexus injuries: mechanism, patterns of injury, and physical diagnosis. *Hand Clinics*.

[23] Horowitz SH (1985). Brachial plexus injuries with causalgia resulting from transaxillary rib resection. *Archives of Surgery*.

[24] Sinow JD, Cunningham BL (1991). Postmastectomy brachial plexus injury exacerbated by tissue expansion. *Annals of Plastic Surgery*.

[25] Luosto R, Ketonen P, Harjola PT, Jarvinen A (1980). Extrathoracic approach for reconstruction of subclavian and vertebral arteries. *Scandinavian Journal of Thoracic and Cardiovascular Surgery*.

[26] Liveson JA, Bronson MJ, Pollack MA (1991). Suprascapular nerve lesions at the spinoglenoid notch: report of three cases and review of the literature. *Journal of Neurology Neurosurgery and Psychiatry*.

[27] Burge P, Rushworth G, Watson N (1985). Patterns of injury to the terminal branches of the brachial plexus. The place for early exploration. *Journal of Bone and Joint Surgery B*.

[28] Nagano A, Ochiai N, Sugioka H, Hara T, Tsuyama N (1989). Usefulness of myelography in brachial plexus injuries. *Journal of Hand Surgery*.

[29] Carvalho GA, Nikkhah G, Matthies C, Penkert G, Samii M (1997). Diagnosis of root avulsions in traumatic brachial plexus injuries: value of computerized tomography myelography and magnetic resonance imaging. *Journal of Neurosurgery*.

[30] Walker AT, Chaloupka JC, De Lotbiniere ACJ, Wolfe SW, Goldman R, Kier EL (1996). Detection of nerve rootlet avulsion on CT myelography in patients with birth palsy and brachial plexus injury after trauma. *American Journal of Roentgenology*.

[31] Volle E, Assheuer J, Hedde JP, Gustorf-Aeckerle R (1992). Radicular avulsion resulting from spinal injury: assessment of diagnostic modalities. *Neuroradiology*.

[32] Brown WF, Veitch J (1994). AAEM minimonograph 42: intraoperative monitoring of peripheral and cranial nerves. *Muscle and Nerve*.

[33] Daube JR, Harprer CM, Desmadt JE (1989). Surgical monitoring of cranial and peripgheral nerves. *Neuromonitoring in Surgery*.

[34] Spinner RJ, Kline DG (2000). Surgery for peripheral nerve and brachial plexus injuries or other nerve lesion. *Muscle Nerve*.

